# How X-ray dark-field imaging relates to small-angle X-ray scattering measurements

**DOI:** 10.1107/S1600576725004017

**Published:** 2025-06-20

**Authors:** Clara Magnin, Manuel Fernández Martínez, Dan Mihai Cenda, Blandine Lantz, Scott Barton, Bertrand Faure, Emmanuel Brun

**Affiliations:** a Univ. Grenoble Alpes, Inserm UA7, Strobe, Grenoble, France; b Xenocs SAS, Grenoble, France; c Xenocs Inc., Holyoke, Massachusetts, USA; Brazilian Synchrotron Light Laboratory, Brazil

**Keywords:** dark-field imaging, small-angle X-ray scattering, multiple refraction

## Abstract

Dark-field (DF) imaging is a recent X-ray imaging modality which is promising because it gives access to information not resolved in conventional transmission X-ray imaging. The DF technique was first introduced as a loss of visibility of the grating interferometry modulations. DF signal is now measured with all the different X-ray phase contrast setups such as beam tracking or modulation-based imaging. Using a dedicated setup [Magnin *et al.* (2023). *Opt. Lett.***48**, 5839–5842], we present in the present article combined measurements of small-angle X-ray scattering and DF signal on the same material. We confirm that DF imaging is sensitive to multiple refraction from a sample, as can be found in the literature on lung imaging, but we show that the DF signal is also sensitive to scattering events. Finally, we measure a porous membrane that creates both types of signal (scattering and refraction), showing that, contrary to existing models, it is difficult to be quantitative about DF.

## Abstract 2

We present the first combined analysis of X-ray dark-field signals and small-angle X-ray scattering curves from several samples representing different sources of scattering.

## 1. Introduction

Conventional X-ray imaging, despite its widespread use, faces significant limitations in terms of spatial resolution. These limitations arise from various factors, including the spatial resolution of the detector, the finite size of the focal spot of the X-ray source, and, in the case of medical applications, the need to minimize the radiation dose or exposure time.

X-ray dark-field (DF) imaging emerged two decades ago (Bech *et al.*, 2010 ▸) as a promising technique to overcome these limitations. By exploiting the scattering of X-rays from micro- and nano-structures, this method can reveal details that are significantly smaller than the pixel size of the X-ray system. DF imaging has been shown to be able to detect early signs of chronic obstructive pulmonary disease (Willer *et al.*, 2021 ▸; Urban *et al.*, 2022 ▸), to detect invisible tooth cracks (Jud *et al.*, 2021 ▸) and to study the microstructure in carbon fibre reinforced materials (Glinz *et al.*, 2022 ▸). Dark-field imaging holds the promise of reducing the acquisition time or the radiation dose required for diagnostic imaging or non-destructive testing.

Historically viewed as a loss of visibility of the modulations obtained in grating-based imaging (Pfeiffer *et al.*, 2008 ▸; Yashiro *et al.*, 2010 ▸), the DF technique is implemented in different experimental setups such as edge illumination or beam tracking (Endrizzi *et al.*, 2014 ▸), analyser-based imaging (Pagot *et al.*, 2003 ▸; Rigon *et al.*, 2007 ▸), single-grid imaging (How & Morgan, 2022 ▸; How *et al.*, 2022 ▸) and more generally modulation-based imaging (Zdora, 2018 ▸; Magnin *et al.*, 2023 ▸). Due to the variety of experimental setups, the physical origin of the DF signal might differ from one setup to another. The common definition of all these techniques is that the DF signal originates from structures smaller than pixels, with contributions from multiple refraction and scattering over a wide range of angles.

Small-angle X-ray scattering (SAXS) and ultra-small-angle X-ray scattering (USAXS) are analytical methods for the characterization of materials on the nanometric scale, consisting in measuring the angle of deviation of X-rays scattered by the sample. SAXS and USAXS can be used to determine the structure of particle systems in terms of the average size or shape and arrangement of the particles within a sample (Glatter, 2018 ▸) over a size range from nanometres to micrometres.

The scattering signal measured in (U)SAXS can be explained from a wave point of view by describing the incident photons as waves of amplitude 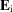 with incident wavevector 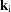. When low-energy X-rays interact with atoms, the dipole formed by the nucleus and its electron cloud begins to oscillate, acting as a secondary radiation source with the same frequency while extinguishing the incident beam. It produces spherical waves 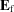 with wavevector 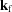 which, due to nanometric differences in electron density within the sample, will interfere along the propagation direction. The interference pattern measured in the detector plane displays variations in intensity *I* as a function of the scattering angle 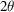. Instead of the scattering angle, the relevant parameter to analyse the interaction is the modulus of the momentum transfer or scat­tering vector 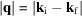, which modulus 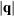 is defined as 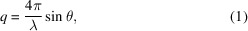
where λ is the wavelength of the incident radiation.

The intensity of the scattered radiation is often represented as an azimuthally integrated 1D diagram as a function of *q*, and thus the measurement of an intensity peak at a given *q* on this diagram allows us to obtain the value of characteristic dimensions or distances *D* in the sample,
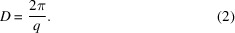


In SAXS, the intensity measured in the detector plane is defined as proportional to the product of a form factor *P*(*q*) and a structure factor *S*(*q*) (Glatter, 2018 ▸; Als-Nielsen & McMorrow, 2011 ▸):
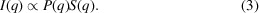
The form factor is defined as the Fourier transform of the pair distance distribution function, characteristic of the particle shape. The structure factor describes interparticle interference and contains information about the interaction between particles. For very dilute systems *S*(*q*) = 1 and can be ignored (Pedersen, 1997 ▸).

The intensity measured in SAXS is also proportional to the square concentration of particles in the volume *V* of the sample. Thus,
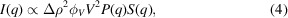
with 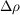 the contrast and 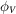 the volumetric fraction.

In this article, we present the very first combined measurement of SAXS and DF signal on the same samples. This study aims to investigate the physical phenomena behind the DF signal by analysing both types of signal.

## 2. Experimental methods

**Figure 1:**
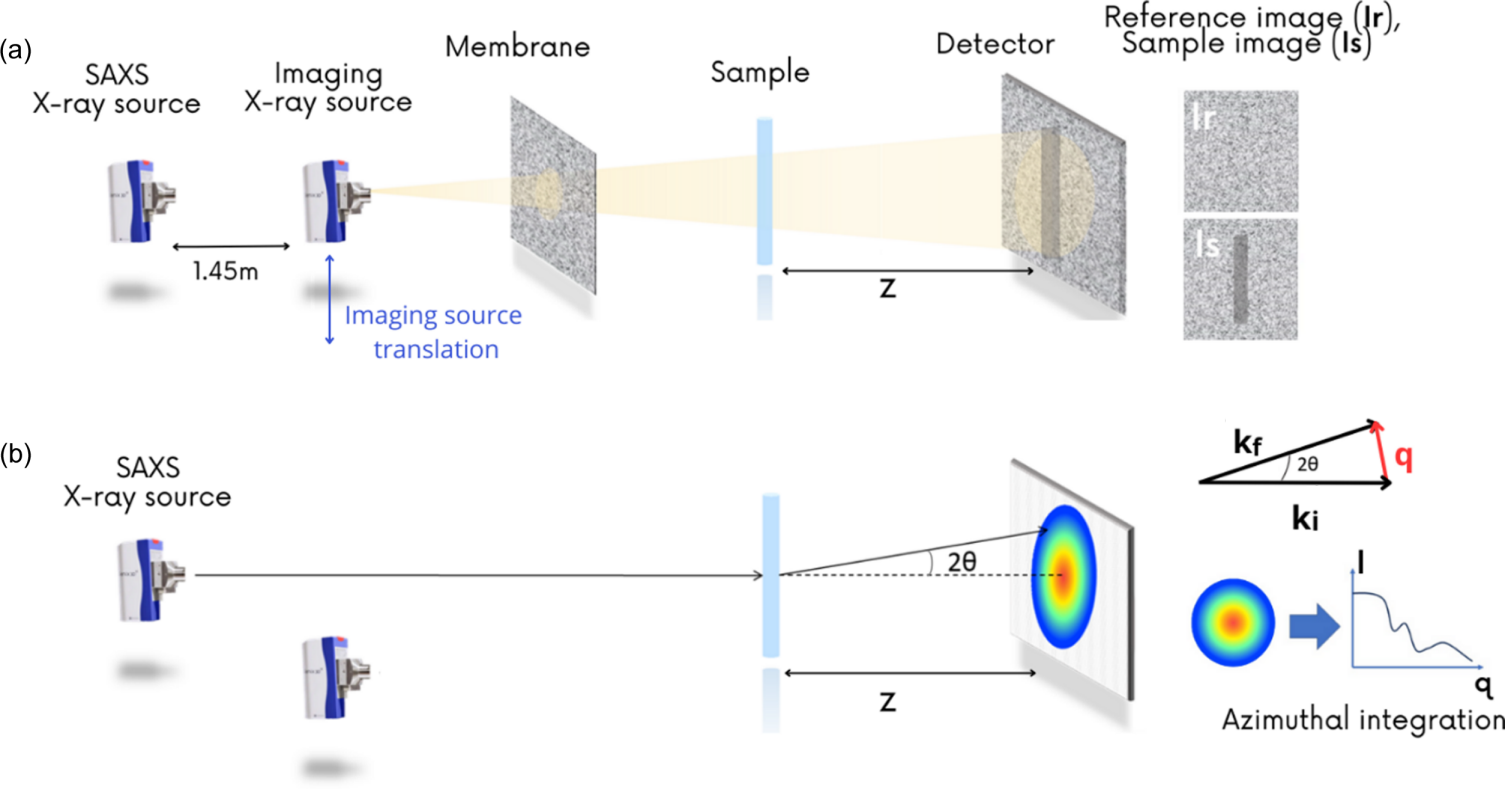
(*a*) Modulation-based imaging experimental setup. A randomly structured membrane placed in the X-ray beam generates random intensity modulations on the images. A first image Ir is acquired as a reference. Without moving the membrane, a second image Is is captured with the sample inserted into the modulated beam. By analysing the distortion of the modulation between the two images one can retrieve absorption, phase, DF and directional DF information. (*b*) SAXS setup. The collimated X-ray beam is scattered by the nanostructures of the sample. The scattering pattern is recorded on the detector and then analysed as a 1D *I*(*q*) curve after azimuthal integration.

The experiments were performed on a laboratory beamline based on a Xeuss 3.0 platform (Xenocs SAS, Grenoble, France) equipped for combined imaging and scattering. The equipment consists of two sources: one monochromatic source with a focused and collimated beam used for 2D SAXS image acquisition, and a second with a cone-shaped polychromatic X-ray beam used for imaging. The system’s SAXS source is fixed, while the imaging source is motorized by a translation motor [Fig. 1 ▸(*a*)]. This motor is used to move the imaging source on and off the sample-to-detector axis. When the imaging source is in the IN position, it is placed in front of the SAXS source and illuminates the sample. When in the OUT position, it is removed from the axis and the SAXS beam can be used.

### 2.1. Modulation-based imaging and SAXS acquisitions

To obtain DF and directional DF images, the modulation-based imaging (MoBI) method, introduced in a previous publication (Quénot *et al.*, 2021 ▸), is used. This method employs a reference membrane that modulates the beam in a random way and relies on the acquisition of pairs of reference (membrane only) and sample (membrane plus sample) images. Once the image pairs are recorded, the LCS algorithm is used to retrieve the DF images (Magnin *et al.*, 2023 ▸).

The experimental parameters of the device are listed in Table 1 ▸ and a schematic of the setup is given in Fig. 1 ▸.

### 2.2. Samples

Three different samples are used in this study:

(i) A glassy carbon sample (GC type T, NIST SRM 3600) from a calibrated batch, 1.9 mm thick, cut into two pieces, half kept in bulk form and the other half crushed into a powder. The powder was roughly ground to obtain large particles and was encapsulated between two layers of Kapton tape to be securely held on the sample holder.

(ii) Five pieces of styrene-butadiene rubber (SBR) of different thicknesses (0.43, 0.51, 0.62, 0.73 and 1.0 mm). SBR is a homogeneous material with a characteristic SAXS signal.

(iii) A sample composed of three anodized aluminium oxide (AAO) nanoporous discs (InRedox, Longmont, USA) with different pore sizes (20, 100 and 160 nm). These discs are all 50 µm thick and 10 mm in diameter. The parameters of these samples are listed in Table 2 ▸.

## 3. Results

### 3.1. Dark field and multiple refraction

An initial step towards understanding DF imaging is identifying the events capable of creating a measurable DF signal. It is often stated that the dark field in imaging is due to multiple refraction phenomena within the sample (How & Morgan, 2022 ▸; Willer *et al.*, 2018 ▸). However, other scattering events caused by the shape, structure and composition of the materials themselves, which are measurable by SAXS, should also contribute to the intensity of the signal in DF imaging. Samples that allow the isolation of possible contributions to SAXS have been experimentally studied to examine their respective impact on the intensity of the DF signal. DF measurements were coupled with SAXS measurements to compare the two signals.

When part of the sample is composed of numerous microstructures that are too small to be directly observed in the image, *i.e.* when the structures are smaller than the system’s resolution, they cause the beam to spread into a cone with a very small angular opening, due to multiple refraction on the surfaces of the microstructures. This average angular opening 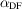 is related to the number of structures *N* encountered by the beam and to the difference in refractive indices of the particles 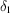 and their surrounding medium 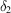 by the relation (von Nardroff, 1926 ▸)
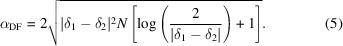
This model has been used repeatedly to interpret DF images of simple samples such as calibrated spheres (How & Morgan, 2022 ▸), as well as more complex ones such as human lungs (Gassert *et al.*, 2021 ▸), where healthy lungs have a relatively high signal due to their many air–tissue interfaces.

A sample of glassy carbon was chosen to verify the assertion that multiple refraction alone can induce a DF signal, as it is known to produce a well defined quantitative scattering signal. A piece of bulk GC sample was used to verify its scattering power, and the GC powder was used to create interfaces that induce multiple refraction, in order to measure the multiple refraction phenomena added to the SAXS signal of the sample.

**Figure 2:**
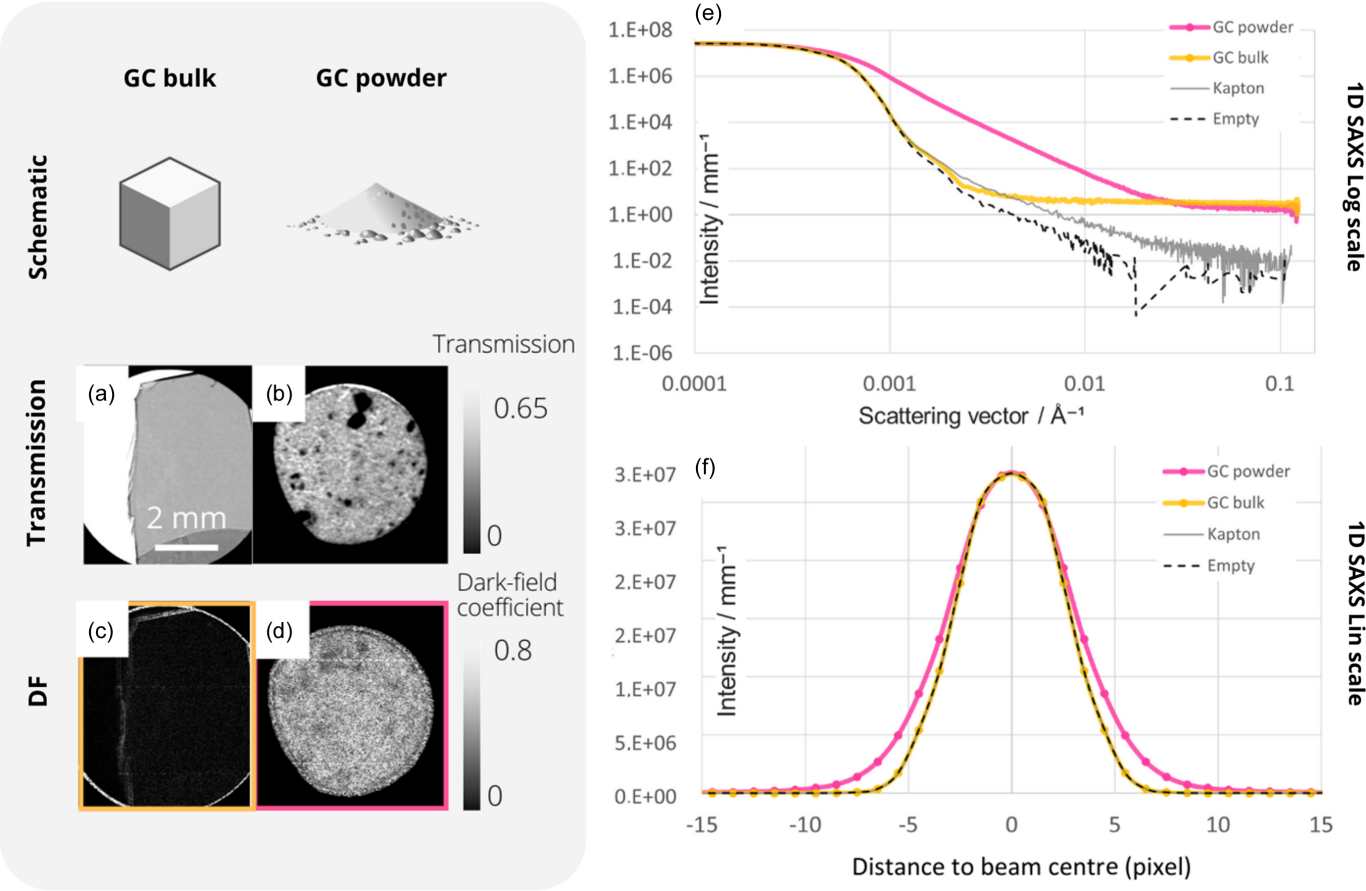
(*a*) and (*b*) Transmission and (*c*) and (*d*) DF (normalized by absorption) images from GC bulk and powder. (*e*) and (*f*) One-dimensional SAXS azimuthal integrations, (*e*) on a logarithmic scale and (*f*) on a linear scale as a function of distance from the beam centre to represent SAXS beam broadening. The sample-to-detector distance is 2500 mm for all measurements.

The results of these measurements are shown in Fig. 2 ▸. The transmission [Figs. 2 ▸(*a*) and 2 ▸(*b*)] and DF [normalized to transmission, Figs. 2 ▸(*c*) and 2 ▸(*d*)] images of the sample obtained using the MoBI method are shown on the left-hand side of the figure. The scattering signals have been analysed and represented in two ways:

(i) On a logarithmic scale in the conventional SAXS form by an azimuthal integration [Fig. 2 ▸(*e*)].

(ii) In a linear mode and using pixel values instead of *q* [Fig. 2 ▸(*f*), which represents the same data as the SAXS plot shown in Fig. 2 ▸(*e*)]. This representation, which shows the intensity profile as a function of distance from the centre of the beam, is possible because the Xenocs setup has no beamstop, allowing direct beam imaging.

In these two SAXS graphs [Figs. 2 ▸(*e*) and 2 ▸(*f*)], the reference curves are shown for the SAXS beam without the sample (‘empty’) and for the SAXS signal through two layers of Kapton tape (‘Kapton’) because the GC powder sample is encapsulated within Kapton tape.

In the transmission images, the average powder transmission is similar to the bulk value (53 ± 2%), since the GC thicknesses are of the same order. However, the DF signals of the two samples are completely different. In the DF images the GC bulk produces a very weak signal (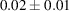) of the order of noise out of the sample (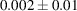), but the powder signal is strong (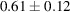). The intensity profile versus distance to beam centre shows a beam broadening in the range of a few pixels for the GC powder, while the bulk GC causes almost no broadening. The 1D SAXS profile shows a clear change in slope from bulk to powder, with a slope in the range of 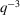 to 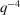 for the GC powder. Here, this characteristic change in slope is mainly attributed to multiple refraction, but total reflection phenomena caused by the powder surface cannot be totally excluded. The respective proportion of these contributions may be a good area to explore. These issues of change in slope have been studied in SAXS in the context of biological tissues or calibrated samples of polymethyl methacrylate powder (Fernández *et al.*, 2002 ▸; Suhonen *et al.*, 2007 ▸). GC in powder form can therefore be used to confirm that multiple refraction, isolated from any other scattering events that may be related to the nature of the sample or its structure, is at the origin of a DF signal that can be measured in imaging. This increase in signal intensity from the GC powder is also observed in SAXS by a significant change in slope between the GC bulk and the powder for *q* ranges from 0.0008 to 0.0012 Å^−1^, as shown in Fig. 2 ▸(*e*).

### 3.2. Dark field in the absence of multiple refraction

Scattering events other than multiple refraction can generate a DF signal even in the absence of interfaces, such as scattering from nanoscale electron-density heterogeneities. In the literature, the DF signal is reported to reflect the SAXS/USAXS signal (Pfeiffer *et al.*, 2008 ▸), and several DF models are based on SAXS theory (Strobl, 2014 ▸; Modregger *et al.*, 2012 ▸; Gkoumas *et al.*, 2016 ▸).

To investigate this hypothesis, DF and SAXS were measured on styrene-butadiene rubber samples, five pieces of different thicknesses. SBR is a homogeneous bulk material, which means that, in the absence of interfaces, no multiple refraction is possible. However, SBR still exhibits a characteristic SAXS signal. Contrary to previous experiments that used sheets of paper in either grating interferometry (Bech *et al.*, 2010 ▸) or beam tracking (Vittoria *et al.*, 2017 ▸), we avoided creating other interfaces than the pure SAXS signal that would explain a nonlinearity between SAXS and DF signals.

**Figure 3:**
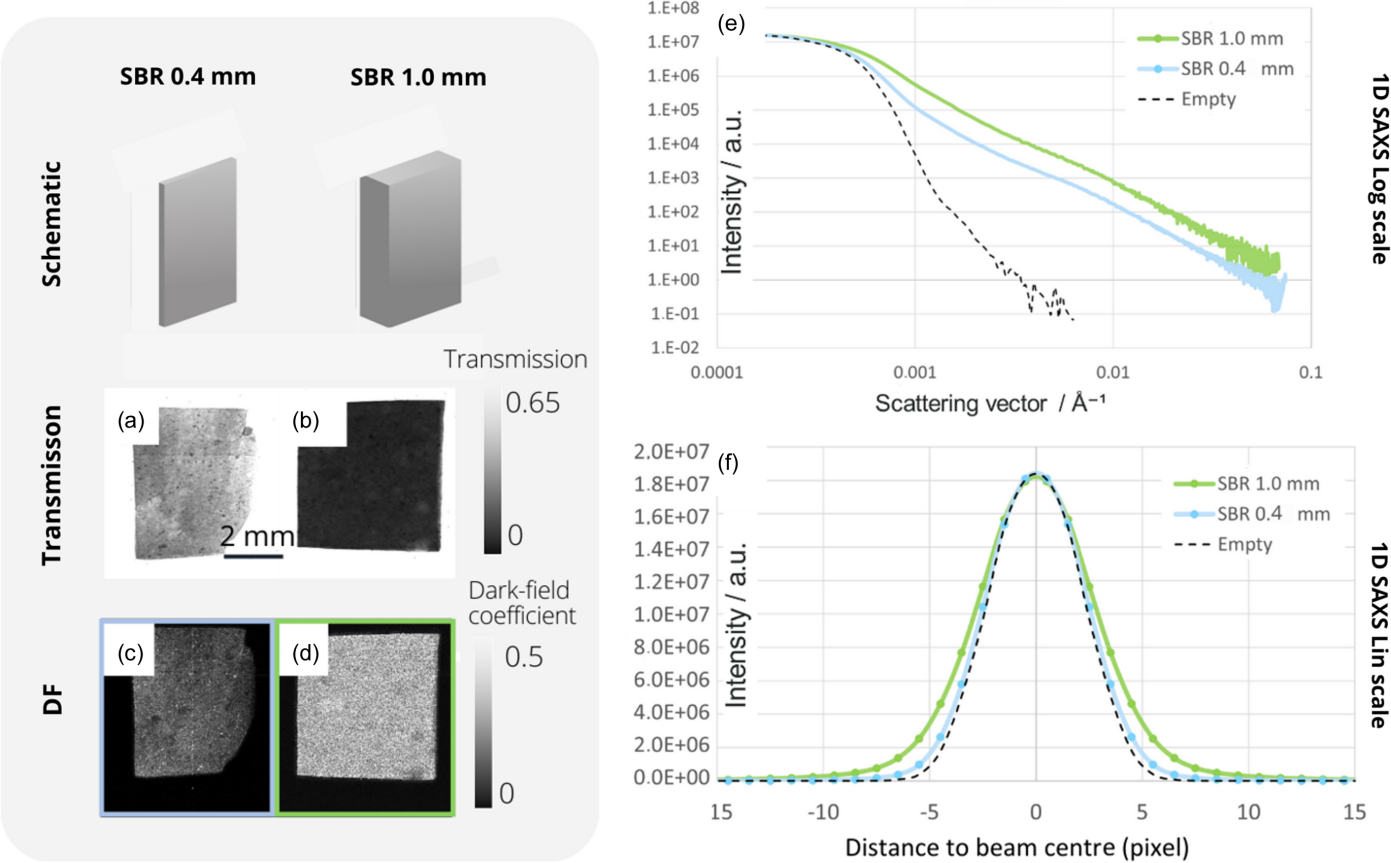
(*a*) and (*b*) Transmission and (*c*) and (*d*) DF images from SBR samples of two different thicknesses. (*e*) and (*f*) One-dimensional SAXS azimuthal integrations, (*e*) on a logarithmic scale as a function of *q* and (*f*) on a linear scale as a function of distance from the beam centre to represent SAXS beam broadening. The sample-to-detector distance is 2500 mm for all measurements.

The results of imaging and SAXS measurements (here not normalized by thickness) obtained on two SBR samples with different thicknesses (0.4 and 1.0 mm) are shown in Fig. 3 ▸. As expected, the absorption (transmission) and DF intensity increase with the thickness of the material. This increase in scattering with sample thickness is also found in the SAXS measurements, with a change in slope and increasing beam broadening in Figs. 3 ▸(*e*) and 3 ▸(*f*), respectively.

**Figure 4:**
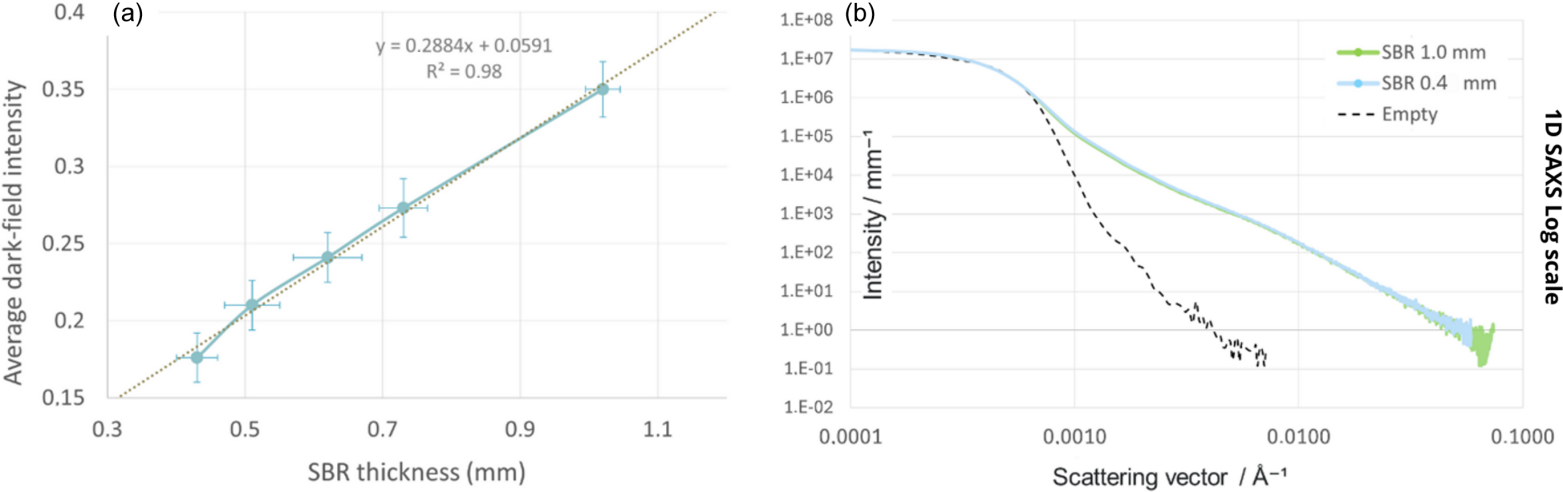
(*a*) Evolution of the DF signal as a function of the SBR thickness and (*b*) 1D SAXS azimuthal integration on a logarithmic scale, intensity normalized to thickness.

To investigate further the impact of material thickness on DF intensity, the evolution of the DF signal as a function of SBR thickness is plotted in Fig. 4 ▸. The thickness maps of the SBR samples are calculated from the transmission images by applying the Beer–Lambert law: 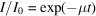, with μ = 23 cm^−1^ being the linear absorption coefficient of the SBR, *t* the thickness of the material passed through, *I* the transmitted intensity and 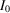 the incident intensity. For each point on the curve, the thickness and average DF intensity are calculated from images of each SBR sample in the same 200 × 200 pixel region of interest.

Fig. 4 ▸(*a*) shows a linear relationship between the thickness of the SBR material and the DF intensity, contrary to what was measured by Vittoria *et al.* (2017 ▸) using a similar method. The overlap of thickness-normalized SAXS intensity curves in Fig. 4 ▸(*b*) confirms this linearity. As this material has no defects or interfaces, only its characteristic SAXS scattering signal contributes to the generation of DF on the image. This analysis shows that multiple refraction is not the only physical phenomenon at the origin of the DF signal, and that equation (5) applies *a priori* in cases where there is no coherent particle or fluctuation scattering, *i.e.* in cases of samples dominated by multiple refraction.

### 3.3. How does SAXS relate to DF in a more complex medium?

The previous samples show a correlation between an increase in the DF signal and the SAXS intensity. To provide a better understanding of the correlation between these two signals, we now extend our study to samples with similar attenuation but different structures on the nanometre scale, which thus produce different characteristic SAXS patterns. Anodized aluminium oxide nanoporous discs, with different pore sizes, were imaged using the MoBI method and measured by SAXS. The sample studied consisted of three AAO discs, each 50 µm thick and 10 mm in diameter, with different pore diameters (20, 100 and 160 nm; Table 2 ▸).

**Figure 5:**
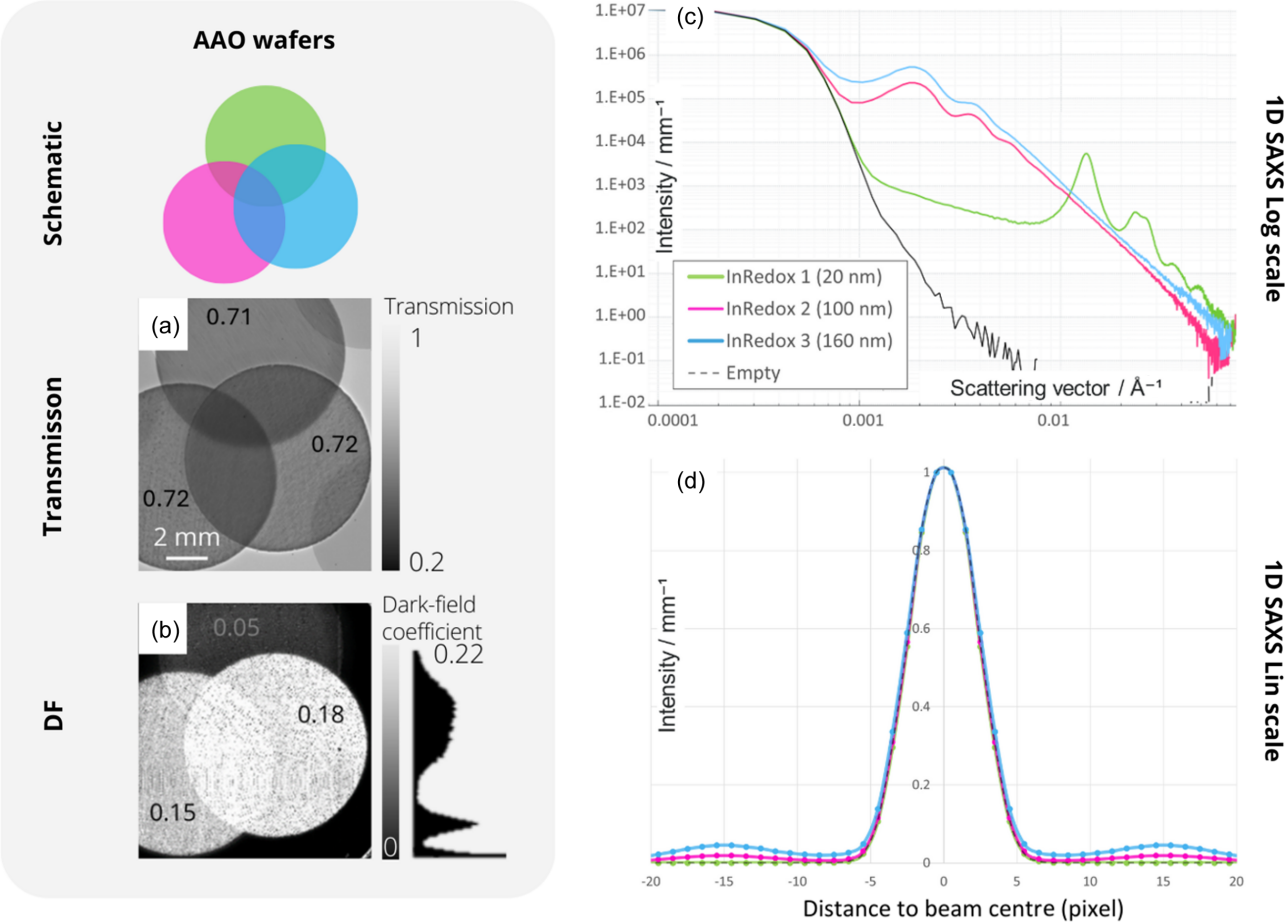
(*a*) Transmission and (*b*) DF X-ray images of AAO wafers with different pore sizes. (*c*) and (*d*) One-dimensional SAXS azimuthal integrations, (*c*) on a logarithmic scale as a function of *q* and (*d*) on a linear scale as a function of distance from the beam centre to represent SAXS beam broadening. The sample-to-detector distance is 2500 mm for all measurements

Due to their similar average porosity (Table 2 ▸), all the wafers have similar transmission (71 ± 0.6%), as shown in the transmission image [Fig. 5 ▸(*a*)]. However, on the DF image [Fig. 5 ▸(*b*)], the signal intensity increases with the pore size of the discs. This first result confirms that the DF signal contains information related to the sub-pixel structures of the sample that are not resolved in conventional transmission imaging.

The SAXS curves obtained for the three samples show oscillations that reflect the characteristic dimensions of the internal nanostructure of the samples [Fig. 5 ▸(*c*)]. The oscillation peaks in discs D2 and D3 indicate the presence of the same characteristic dimension related to a form factor *P*(*q*) at *q* ≃ 0.002 Å^−1^, corresponding to a dimension *D* ≃ 314 nm according to equation (2). The intensity peak of the D1 curve at *q* ≃ 0.014 Å^−1^ suggests a characteristic size of 45 nm. These values are not consistent with the pore sizes indicated by the manufacturer (Table 2 ▸) for the three discs. Taking into account the diameters reported, wafers D1, D2 and D3 should exhibit oscillations at *q* values of approximately 0.031, 0.006 and 0.004 Å^−1^, respectively. These deviations from the expected values may be attributed to inadequate calibration of the pores or the possibility that the characteristic dimensions measured by SAXS represent the distances between the pores rather than the actual pore size.

First of all, the SAXS intensity profile in Fig. 5 ▸(*d*) shows no broadening of the beam around its centre of mass. The intensity variations are localized on pixels further away. Therefore, the DF signal measured from these discs cannot be explained by the broadening of the direct beam at very small angles or by existing models based on Gaussian broadening (Bech *et al.*, 2010 ▸; Wang *et al.*, 2009 ▸; Croughan *et al.*, 2023 ▸; Buchanan *et al.*, 2023 ▸; Esposito *et al.*, 2023 ▸).

Comparing next the SAXS signal with the DF intensity, the DF signal from the three wafers [Fig. 5 ▸(*b*)] increases in line with the SAXS curves [Fig. 5 ▸(*c*)] at low *q* values (below 0.01 Å^−1^). Indeed, for the *q* range from 0.0006 to 0.01 Å^−1^, the SAXS and DF scattering intensities for the three discs are in agreement and show that disc D3 has a higher scattering intensity than disc D2, which in turn is higher than disc D1.

However, when considering higher *q* values (*i.e.* higher scattering angles), this relationship is no longer valid: the intensity from disc D1 becomes higher than that of the other samples in the SAXS curve (intensity peak at 0.03 Å^−1^) and the DF image of D1 is the darkest. This can be explained in several ways. On the one hand, this result may indicate that the dark field is not sensitive to the intensity peak of D1 because it occurs at too high a scattering angle. According to this hypothesis, the dark field would have a sensitivity range of *q* < 0.01 Å^−1^. Therefore, on the basis of these observations, the DF images in this imaging configuration and at an energy of 8 keV would reflect scattering events occurring at angles θ < 1.23 mrad [equation (1)], which belong to the USAXS domain. On the other hand, the DF signal might represent the area under the SAXS curve. In this case, it could be sensitive to higher *q* values than in the previous assumption and this could explain why the DF intensity for the discs follows the order D3 > D2 > D1. This hypothesis also explains why the DF intensity measured for disc D1 is not zero, as the intensity of the peak at *q* < 0.014 Å^−1^ would contribute to the signal. Both hypotheses are consistent with the results obtained from the GC sample (Section 3.1), where the DF intensities in the imaging are in agreement with the SAXS measurements, taking into account the area under the SAXS curves but also considering a range of *q* < 0.01 Å^−1^.

## 4. Discussion and conclusion

In this study, SAXS and DF measurements were conducted concurrently on the same sample using a single X-ray device (Xeuss 3.0, Xenocs SAS). This combined analysis of both signal types across various samples revealed several properties of the DF signal measured through the MoBI imaging method. Given the very similar experimental parameters (energy, distances, pixel size) we can assume that the scattering angular sensitivity is the same for both SAXS and DF imaging.

Firstly, the role of multiple refraction in generating a measurable DF signal in imaging was verified using a sample where the sole source of X-ray scattering was multiple refraction. This result confirms the significance of multiple refraction in DF signal generation, as demonstrated in several previous scientific studies (How & Morgan, 2022 ▸; Willer *et al.*, 2021 ▸; Willer *et al.*, 2018 ▸).

Furthermore, it was shown that contributions other than multiple refraction, such as those related to the material’s nanostructure (multiple scattering), can also generate a DF signal, even in the absence of interfaces within the sample. This indicates that the DF signal is sensitive to a broader range of scattering events than previously thought.

Finally, combined SAXS/DF measurements on nanoporous discs established that the DF signal reflects events measured by SAXS, at least for certain *q* values within the USAXS domain. This result opens up interesting possibilities of using DF imaging for rapid mapping of the distribution of scattering intensities within a sample.

The ensemble of these measurements raises questions about the validity of simple DF models, particularly the one described by von Nardroff [equation (5)], when applied to complex samples such as lungs (as mentioned in Section 3.1). In samples with structural features in the nanometre to micrometre range, the effects of multiple refraction cannot be entirely isolated from other scattering phenomena that may contribute to DF generation. Indeed, if the DF intensity is equivalent to that measured by SAXS methods for certain angular ranges, then modelling the dark field becomes complex. It necessitates considering all sample parameters capable of influencing the spatial distribution of electron density, such as composition, shape and size, from the microscopic to the molecular scale.

The main limitation of this study is that we relied on a single experimental setup to measure the dark field. Modulation-based imaging is the simplest technique to implement experimentally, but the same numerical techniques can be used in grating interferometry (Zdora *et al.*, 2018 ▸). Further experiments using other experimental configurations are foreseen.

Further analyses are underway to determine the angular sensitivity range of DF with better accuracy. By varying parameters such as wavelength, propagation distance or modulation size, DF images at different scattering angle intervals could be obtained, thereby probing different *q* ranges. Therefore these results pave the way for future research aimed at refining DF imaging techniques and expanding their applicability to a broader range of complex biological and material samples.

## Figures and Tables

**Table 1:** Experimental setup parameters

Distances	Source to sample 0.55 m, sample to membrane 0.1 m, sample to detector (*z*) 2.500 m
SAXS beam	GeniX3D X-ray generator, copper anode, 50 kV, monochromatic at 8 keV (Cu *K*α_1,2_), collimated beam, no beamstop
Imaging beam	X-ray generator, copper anode, 30 kV, polychromatic at 8.6 keV, average energy, cone-shaped beam
Detector	Photon-counting Eiger2 hybrid pixel (DECTRIS, Switzerland) with pixels of 75 µm, imaging acquisition time 30 s per image, SAXS acquisition time 600 s
Membrane	Piece of sandpaper, grit size P320

**Table 2:** AAO InRedox wafer parameters

Disc number	Pore density (cm^−2^)	Porosity (%)	Pore size (nm)
Disc 1	6 × 10^10^	12	20
Disc 2	1 × 10^9^	12	100
Disc 3	1 × 10^9^	20	160
